# 53 Use of Polylactic-acid-membrane in Split-thickness Skin Graft Donor Sites: A Prospective, Comparative, Randomized Study

**DOI:** 10.1093/jbcr/irac012.056

**Published:** 2022-03-23

**Authors:** Nicholas Möllhoff, Denis Ehrl

**Affiliations:** Division of Hand, Plastic and Aesthetic Surgery; University Hospital, LMU Munich; Germany, Munich, Bayern

## Abstract

**Introduction:**

Polyurethane film (PU) dressings are commonly applied for coverage of split-thickness skin graft (SSG) donor sites, while previous studies have suggested reduced morbidity using a polylactic acid membrane (PLM). To further investigate the optimal treatment approach, the presented study compared outcome of donor sites in patients receiving either PLM or PU.

**Methods:**

This randomized clinical trial allocated patients requiring SSG to receive either PLM or PU at the donor-site. Primary endpoint was difference in donor site scar appearance between groups 3 months postoperatively (Vancouver Scar Scale – VSS). Secondary endpoints included pain, the number of and time required for wound dressing changes, and costs related to the wound dressing.

**Results:**

30 patients were allocated to each group. The median VSS scored lower for patients receiving PLM (PU: 3 (Q1: 2; Q3: 4) vs. PLM: 2 (Q1: 1; Q3: 3); p=0.049). Pain during change of wound dressing (PU: 2.0 ± 0.2 vs. PLM: 0.5 ± 0.2; p< 0.001) and mobilization (PU: 0.8 ± 0.2 vs. PLM: 0.3 ± 0.1; p=0.032) was reduced in the PLM group. Patients with PLM required less dressing changes per day of hospital stay (PU: 0.44 ± 0.06 vs. PLM: 0.28 ± 0.02; p=0.015). Mean time for wound dressing changes per patient was higher in the PU group (PU: 74.50 ± 5.72 vs. PLM: 21.43 ± 2.61 min; p< 0.001). Costs were higher in the PLM group (PU: 67.83 ± 5.56 vs. PLM: 162.79 ± 21.76 €; p< 0.001).

**Conclusions:**

PLM improves outcome of SSG donor sites, however, higher treatment costs must be taken into consideration.

